# Shaping markets to benefit global health – A 15-year history and lessons learned from the pentavalent vaccine market

**DOI:** 10.1016/j.jvacx.2019.100033

**Published:** 2019-07-18

**Authors:** Melissa Malhame, Edward Baker, Gian Gandhi, Andrew Jones, Philipp Kalpaxis, Robyn Iqbal, Yalda Momeni, Aurelia Nguyen

**Affiliations:** a Gavi, The Vaccine Alliance, Global Health Campus, Chemin du Pommier 40, Grand-Saconnex 1218, Switzerland; b UNICEF Supply Division, Oceanvej 10-12, Copenhagen 2150, Denmark; c The Bill & Melinda Gates Foundation, 500 Fifth Ave. North, Seattle, WA 98109, USA

**Keywords:** Market shaping, Global health, Vaccines, Immunisation, Pentavalent, Market dynamics, US$, United States Dollar, B, billion, BMGF, The Bill & Melinda Gates Foundation, DALY, disability-adjusted life-years, DTP, diphtheria tetanus pertussis vaccine, DTwP, diphtheria tetanus whole-cell pertussis vaccine, Gavi, Gavi The Vaccine Alliance, HepB, hepatitis B vaccine, Hib, *haemophilus influenzae* type b vaccine, HMF, Healthy Market Framework, IPV, inactivated polio virus, M, million, MIC, middle-income country, NRA, National Regulatory Authority, PAHO, Pan American Health Organization, Roadmap, Supply and Procurement Roadmap, PRG, Procurement Reference Group, SPS, Supply and Procurement Strategy, WAP, weighted average price, WHO, World Health Organization

## Abstract

Market shaping for health products used in lower-income countries strives to benefit public health. As a funder of vaccines, Gavi, The Vaccine Alliance (Gavi) has goals for its market shaping efforts, achieved through a strategy developed and implemented by the Gavi Secretariat, UNICEF, the World Health Organization (WHO) and the Bill & Melinda Gates Foundation (BMGF). A case-study of Gavi’s fifteen-year engagement with a vaccine against diphtheria, tetanus, pertussis, hepatitis B and *haemophilus influenzae* type b (pentavalent) provides evidence of the benefits and potential risks of trying to influence markets. During 2001–18, Gavi disbursed US$3.5 billion to support use of 50 million pentavalent doses annually before 2005, increasing to ∼300 million doses annually by 2016. During this time, eight manufacturers invested in vaccine development and manufacturing and the first two manufacturers have subsequently ceased production. Following its strategy, Gavi implemented coordinated market interventions including technical assistance to manufacturers, improving market information transparency, risk-sharing agreements and innovative procurement aiming to stimulate and capitalize on a competitive market. In 2018 supply allows ∼80 million children per year to be immunised, a sixteen-fold increase from 2005, with vaccine-related costs per child for donors and countries of one-quarter the 2005 level. Lessons learned include the importance of frameworks and strategies; the need to adjust interventions with changing conditions; the important role of manufacturers; and the potentially powerful effects of interconnected markets. This case study is limited by its focus on a single health product in a specific market, however the lessons can inform other market shaping efforts when taken in context. While countries and children have improved vaccine access, risks of financial sustainability and continued manufacturer investment in Gavi vaccine markets are being monitored. Gavi should continue implementing a market shaping strategy, adjust with market conditions and expect and measure unintended consequences.

## 1 Introduction

Market failures for vaccines and other health products for lower-income countries frequently emerge where mismatches between demand and supply lead markets to function sub-optimally [1], [2], [3], often constraining public health program implementation. Strategies seeking to address these market shortcomings have been implemented through Gavi, the Vaccine Alliance (Gavi) [2], [3], [4], [5] and have generated tangible benefits. The use of market influencing strategies for public health products is relatively new and there are few instances where the effects of these strategies have been observed and characterised over time. This case study of the market for a vaccine containing diphtheria, tetanus, whole-cell pertussis vaccine (DTwP), hepatitis B vaccine (HepB) and *haemophilus influenzae* type b vaccine (Hib), referred to as “pentavalent”, describes the market environment and market shaping actions from 2005 and projected to 2020. It offers lessons learned and provides the opportunity for funders, market influencers and manufacturers of health products to recognize and act on lessons from this experience.

### 1.1 The importance of pentavalent

Diphtheria, tetanus, pertussis vaccines (DTP) have been used since the 1950s and from the 1990s were combined with HepB and/or Hib and shifted to contain an acellular pertussis component in higher-income countries [6]. Before 2000, the lack of disease burden awareness, financial constraints and the poor suitability of these vaccines for lower-income countries limited use of HepB and Hib [2], [7]. In 2000 less than 10% of the target population in the African region of the World Health Organization (WHO) were vaccinated with HepB and Hib, while more than 50% were vaccinated with DTP [8]. Starting in 2000, Gavi’s funding and building awareness of the benefits of Hib transformed demand for this family of vaccines [7], [9], [10] and attracted interest from emerging manufacturers for pentavalent. The market for pentavalent containing a whole-cell pertussis component, delivered as a course of three doses per child, is exclusively in lower-income countries and as such, manufacturer revenues are limited to lower-income countries.

By 2017, more than 70% of the same target population were vaccinated with DTP, HepB and Hib, primarily using pentavalent, increasing the number of Hib-vaccinated children by >20 M annually compared to 2000 [8]. Pentavalent is estimated to avert the largest number of deaths and disability-adjusted life-years (DALYs) when considering vaccination against 10 diseases in 73 Gavi-supported countries [11]. From 2001 to 20, it is estimated that 10 M deaths and 390 M DALYs will be averted and more than US$250B in economic and social value generated from only the Hib and HepB components in 73 Gavi-supported countries [11].

### 1.2 Vaccines and market shaping

**Fig. 1:**
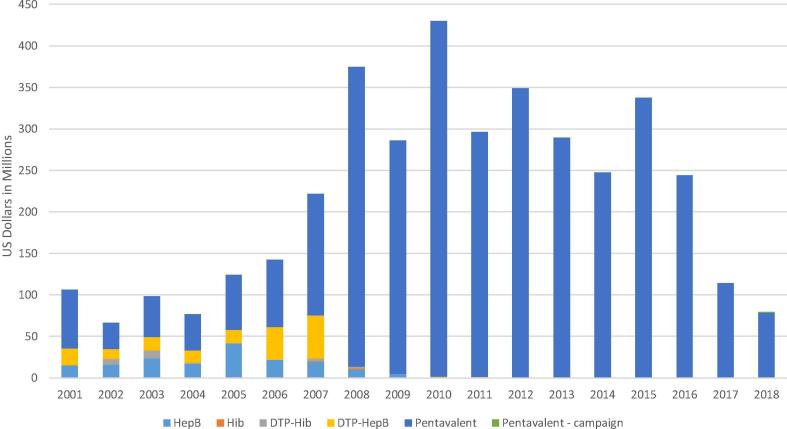
Annual disbursements by Gavi for pentavalent and related vaccines, 2001–18 [13].

Gavi’s primary purpose is to increase the uptake of vaccines and to reduce the historical delay of 15–20 years for new vaccines to reach developing countries [4]. From its formation, it has engaged vaccine manufacturers as partners through representation on its governing body [12]. Gavi disbursed US$9.3B for vaccine procurement, including US$3.5B for pentavalent procurement from 2001 to 18 [13] (Fig. 1). These funds are channelled predominantly through UNICEF and the Revolving Fund of the Pan American Health Organization (PAHO), that each provide a centralised, strategic procurement approach on behalf of countries and benefit from prequalification by WHO to ensure that vaccines meet safety, efficacy and programmatic suitability requirements [3], [5], [14].

Gavi’s model was initially to harness market forces and influence the availability and pricing of vaccines by providing funding, generating and organizing demand from countries, and using centralised procurement to increase market influence [15]. This approach built on the prior and long-term experience of UNICEF and its 2002 Vaccine Security Strategy, to approach vaccine markets for the poorest countries differently (e.g. through use of longer term- and firm- contracts to improve manufacturer’s visibility of demand and reduce associated risks, and to move beyond price considerations alone) when contracting supply [16]. In 2004, Gavi adopted procurement principles of vaccine security, affordable supply, and transparent product-specific procurement strategies [17]. A 2010 evaluation noted that Gavi had been successful in generating demand, but that supply strategies had not yet sufficiently improved supply stability or affordability of pentavalent as compared to the Alliance’s initial expectations and ambitious goals [12].

**Fig. 2:**
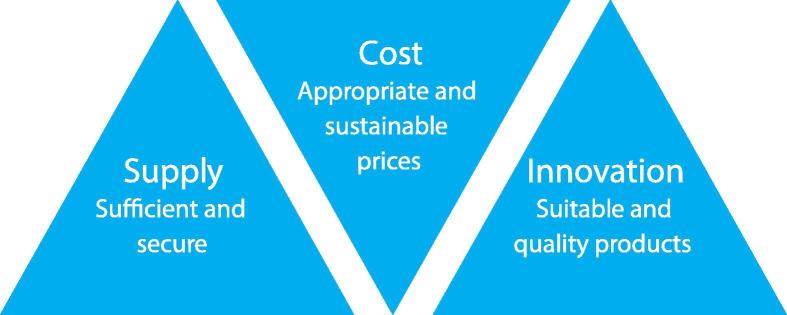
Priorities of the Supply and Procurement Strategy [3].

In 2011, Gavi introduced a strategic goal to “shape the market for vaccines and other immunisation supplies” and revised its Supply and Procurement Strategy (SPS) [3], [4], [10]. The new focus was based on accumulated experience and greater understanding of market characteristics including; significant upfront investments, long development and manufacturing lead times, continued uncertainty and volatility in demand for many vaccines, the relatively small potential revenue generated by vaccine sales in low-income compared to high-income countries, and a dearth of market information. The SPS established priorities on supply, costs and innovation (Fig. 2), all supported by increasing information transparency [3], [5]. The SPS acknowledged the potential tensions among objectives and directed them to be resolved on a vaccine-by-vaccine basis [3]. Alliance partners that implement the strategy are Gavi Secretariat in a coordinating role, procurement partners UNICEF and PAHO managing the majority of procurement, supply and distribution for funded vaccines, the WHO developing product specifications and standards, manufacturers providing supply of quality and affordable vaccines, and the Bill & Melinda Gates Foundation (BMGF) providing financial and technical support to select manufacturers [5].

Tools used to implement the SPS include Supply and Procurement Roadmaps (Roadmaps) for each vaccine which describe market information, provide the platform to consider different strategic options and balance priorities and then establish and coordinates strategies and interventions among market shaping partners and are a tool for transparent communication to manufacturers [3]. UNICEF develops and implements procurement strategies that support the market strategy to contribute to vaccine security and healthy markets and since 2006 has used Procurement Reference Groups (PRGs) to improve coordination and transparency in the procurement process and to access additional expertise in strategic procurement considerations. Supporting information transparency, reducing information asymmetries and improving market functioning, UNICEF publishes price and market analyses and hosts annual consultations with manufacturers [18].

**Fig. 3:**
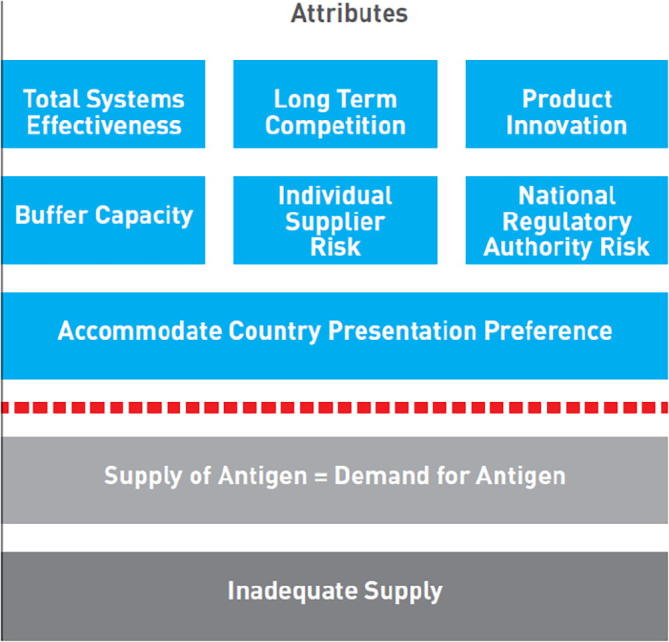
The Healthy Markets Framework [19].

The Healthy Market Framework (HMF), created in 2015, articulates “market health” and desired market outcomes, and helps market shaping partners to weigh the trade-offs between various market attributes [5] (Fig. 3). As part of the HMF, costs are assessed per attribute, allowing quantification of the investment needed to achieve a healthier market or the savings that can be achieved [19].

Following an evaluation of the SPS and its implementation, an updated SPS for 2016–20 supplemented the initial priorities with strategic priorities to deliver results based on a transparent definition of a “healthy market” using the HMF, to take a long-term view of markets including identifying the point when a market no longer requires market shaping interventions, monitoring unintended consequences of market shaping activities, and to improve support for product innovation [5]. These strategic priorities will be assessed after 2020 [5].

## 2 The case study of Pentavalent: Sources and results

**Fig. 4:**
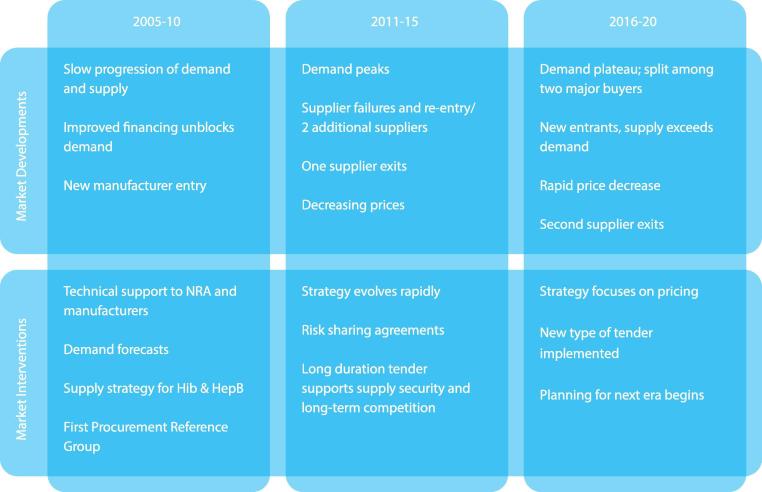
Summary of pentavalent market development and interventions, 2005–20.

Pentavalent is the first Gavi-supported market to reach fully satisfied demand and where results of multiple interventions and market response can be evaluated. The period 2005–20 was chosen because it represents the period of demand growth, market shaping activity and a view to the near future (see Fig. 4).

### 2.1 Information sources

This case study relies on published data, while the discussion and conclusions are primarily based on the personal experiences of the authors whose organizations planned and implemented the strategies and market interventions described. Public sources are cited whenever possible; however, the authors’ opinions have been informed by additional commercially sensitive information not available publicly. Interviews with four individuals and the six currently active manufacturers supplemented the authors’ knowledge and helped provide a manufacturer viewpoint to augment the other perspectives.

### 2.2 Results

#### 2.2.1 A measured, steady start: 2005–10

**Fig. 5:**
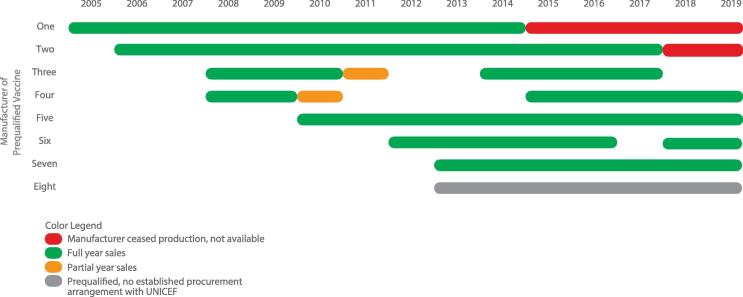
Prequalified pentavalent and years of sales to UNICEF.

**Fig. 6:**
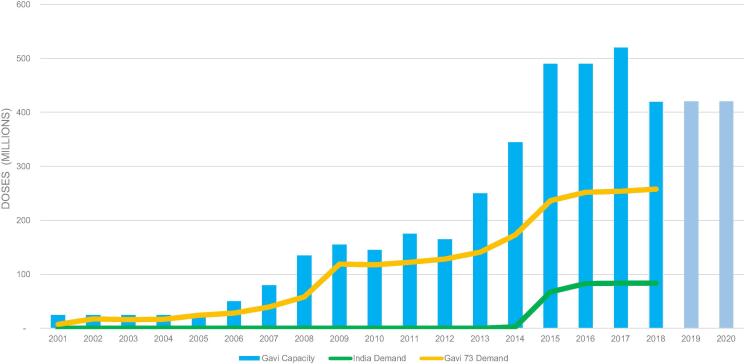
Demand and manufacturing capacity of prequalified pentavalent, 2005–20.

**Fig. 7:**
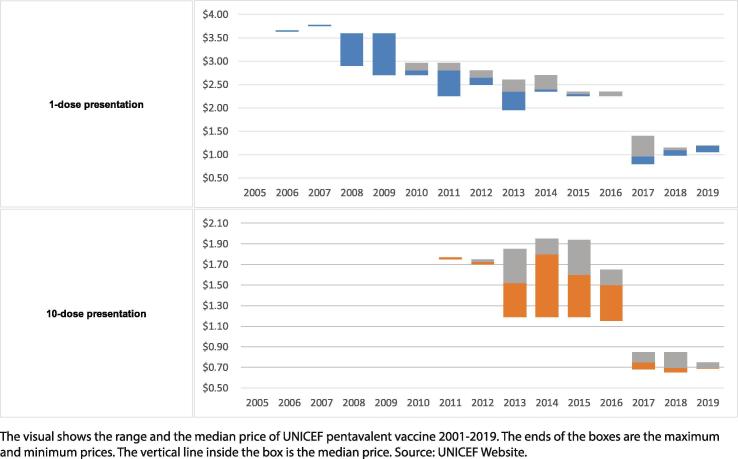
Pentavalent per dose price ranges to UNICEF, 2005–19.

The market developed steadily with demand increasing from <50 to ∼100 M doses, triggered by increased country demand and donor investments through Gavi [20], [21] (Fig. 6). Manufacturers supplying prequalified vaccine increased from one to five, including three based in India [20] (Fig. 5). Starting in 2010, supply entered a volatile period [22] after one new vaccine lost prequalified status [23]. Triggered by new entrants, prices decreased from ∼US$3.60 in 2005 to US$3.20–2.25 per dose (US$9.60–6.75 per course) by 2010 [24] (Fig. 7).

Investments of emerging manufacturers during this period was considerable and included sourcing of component vaccines, technology transfers, clinical and analytical development, registration and prequalification activities as well as building manufacturing capacity to meet increasing demand. Some manufacturers report individually investing >US$100 M over three to ten years [25] and several benefitted from co-investments of the BMGF.

The first product specific supply strategy for Hib and HepB in 2005 called for better coordination of partners and consistent communication to manufacturers [17]. Accordingly, a PRG worked with UNICEF and partners to create a procurement strategy and to monitor outcomes for 2007–09 [26]. The PRG was guided by procurement objectives enshrined in the strategy: a healthy market, ensuring the sustainable quantity of supply through a diverse supplier base; select products the best meet country needs and; achieve a long-term affordable price [26]. Increasing market information and certainty for manufacturers began through publication of a Strategic Demand Forecast by the Gavi Secretariat, UNICEF’s publication of price data and a product menu [27] and continued use of longer-term procurement arrangements with suppliers [3], [10], [12], [17]. Technical assistance implemented by WHO for National Regulatory Authorities (NRA) to meet standards for vaccine regulation and for manufacturers to meet standards for prequalified vaccines was accelerated [14].

#### 2.2.2 Rapid change and market development: 2011–15

The market developed rapidly and annual demand for pentavalent reached > 200 M doses by 2015 [22] (Fig. 6). Through 2012 supply was volatile and barely met demand; a second vaccine lost prequalification status [28] and others experienced interrupted supply [22]. Supply evolved rapidly with the two vaccines that had lost prequalification status regaining it by 2014, two new vaccines from manufacturers in India and Korea entering, and the original manufacturer ceasing production [20] (Fig. 5). By 2015, seven prequalified vaccines were available [20] and a lower cost multi-dose vial packaging became widely used. By 2015 prices were US$2.35–1.19 per dose (US$7.05–3.57 per course) [24] representing a range of packaging (Fig. 7).

Market interventions including technical support by the WHO to NRAs and manufacturers [14], technical assistance to several manufacturers, facilitated through PATH and funded by the BMGF, intended to increase production efficiencies and improve production economics, primarily for the older manufacturing methods for DTwP [25] accelerated during this period.

The rapidly evolving supply challenges required significant short-term interventions to ensure continuous availability in countries. UNICEF closely monitored vaccine availability at country level and avoided interruptions to vaccination. To help manage the changing market situation, and increase shared understanding, UNICEF published market notes and continued regular strategic dialogues with manufacturers through its annual industry forums [18].

In 2012 BMGF executed a risk-sharing agreement that helped bring a new manufacturer and additional supply into the market. BMGF funded technical support to the manufacturer while simultaneously establishing a price point ∼35% lower than the next lowest available to UNICEF through a volume guarantee executed in parallel to the UNICEF tender framework [24]. This agreement generated savings of ∼US$150 M over four years [29] and increased understanding of potential levels of sustainable pricing for some manufacturers. Designed to last for five years, the volume guarantee ended one year early due to significantly changed market conditions [30].

During 2013, the first Roadmap for pentavalent predicted demand, including India, reaching 300 M doses annually and six to eight manufacturers in the market by 2016. The strategy called for ensuring reliable supply and continued price decreases to a “low but sustainable level for both countries and manufacturers” [31].

UNICEF supported the strategy through a strategic tender for supply during 2013–16 structured to bring stability to a rapidly growing market which had just recovered from supply challenges [32]. When completed in early 2013, the tender anticipated entry and re-entry of manufacturers and provided a means for them to gain sales as their vaccines became prequalified. A retrospective review of the 2013–16 tender showed that this approach improved supply security and long-term competition and cost US$0.15 per course more than a cost minimizing approach [5]. Pricing for the tender period of US$2.95–1.15 per dose (US$8.85–3.45 per course) [24] represents a range of packaging and special terms associated with the lowest prices.

A second Roadmap published in 2014 reflected the rapidly evolving market including having reached a theoretical annual capacity of > 400 M doses, concentrated in three manufacturers (Fig. 6) [22], [33]. It recognized the opportunities to decrease prices during 2015–16 inherent in India’s decision to fully utilize pentavalent, leading to assured demand of ∼300 M doses annually, and increasing competition. The Roadmap called for significant price reductions for Gavi-funded vaccines from 2017. It recognized that the market evolution might include inactivated polio virus (IPV) vaccine as part of a combination vaccine and the importance of this to manufacturers and market shapers [33].

In 2014, noting that offered pricing for fully self-funded, middle income countries (MICs) was on par with Gavi-funded countries [34], UNICEF and the Gavi Secretariat secured a price decrease through special contracting terms within the 2013–16 tender period [22], [35]. The price decrease generated savings of > US$50 M over 2015–16 [36] and leveraged UNICEFs engagement in MIC demand [25].

Finally, during 2015 the first significant procurement from an entity other than UNICEF began. The Government of India, whose volume represents approximately one-third of the global total [22] (Fig. 6), executed a two-year tender with annual demand of ∼90 M doses reported at a value of US$133 M, resulting in prices lower than available to UNICEF at the time, albeit under different contracting terms [37].

#### 2.2.3 A new era: 2016–20

Demand peaked and reached a steady state of ∼300 M doses annually [22] (Fig. 6). Procurement is slightly less consolidated because India has emerged as a second major buyer and there is the potential for countries previously supported by Gavi to self-procure. In 2016, there were seven prequalified vaccines (Fig. 5), representing ∼600 M doses of theoretical annual manufacturing capacity (Fig. 6) and a strong recent history of reliable supply. By 2018, four manufacturers were supplying UNICEF, two were supplying locally and a second had ceased production of pentavalent [22], [25]. Pricing to UNICEF declined significantly and in 2019 reached US$1.20–0.69 per dose (US$3.60–2.07 per course) for different packaging [38] (Fig. 7).

Interventions in this period were driven by a Roadmap, developed in 2016 and published in 2017, that recognized the context of plateaued demand, estimated manufacturing capacity that significantly exceeded demand, integrated outcomes from the Government of India tender and implemented the HMF principles for the first time. The Roadmap called for maintaining a diversified supplier base and further price reductions in the next UNICEF tender. Additional considerations reflected the desire to maintain availability and low pricing for countries newly self-funding procurement [39].

During 2016 UNICEF developed a procurement strategy in consultation with partners and manufacturers, leading to its first phased tender. This tender modality was designed to harvest the benefits of competitive dynamics in a fair and transparent manner [40] and allow market competition to set prices through manufacturers taking informed pricing decisions. For manufacturers it increased price transparency, including an opportunity to gauge competition and adjust their strategies in a second phase, avoiding the potential to mis-read the market and lose the prospect for any sales during the period. Tender objectives included consideration to preference manufacturers that have a pipeline of vaccines targeting UNICEF/Gavi markets [40]. The tender for supply during 2017–19 included demand for countries transitioning from Gavi and non-Gavi MICs procuring through UNICEF [40].

Prices published after the first phase and representing a portion of the volume, were US$1.40–0.75 per dose in different packaging [41]. The price discovery enabled through the first phase allowed manufacturers to adjust their pricing strategy for the second phase with prices as low as US$0.60 per dose. Full tender outcomes included agreements to procure ∼150 M doses annually from six manufacturers at US$1.40–0.60 per dose in different packaging (US$4.20–1.80 per course) [24]. These prices are expected to provide saving of more than US$350 M for Gavi-funded procurement [30], [39].

During the second phase, one manufacturer announced that it would cease pentavalent production and a second did not decrease its initial price offering, both citing unsustainable business at prevailing prices. As a result of the tender, six manufacturers supplied in 2017 and four are supplying in 2019 [22] (Fig. 5).

During 2017–19, the same prices were offered by manufacturers to UNICEF for countries not supported by Gavi [22]. This solidified the trend toward equivalent pricing for countries as the market became more competitive.

A 2018 news report indicates that in the Government of India’s second procurement of pentavalent for fiscal years 2019–20, it will buy pentavalent at a lower price than previously, with the majority sourced from a local manufacturer with a non-prequalified vaccine [42].

Updating the Roadmap to reflect new market conditions including results of the second Indian tender and planning for demand from 2020 are underway. This planning will incorporate lessons, consider all market developments, Gavi’s new vaccine investment decisions, healthy market objectives and the potential evolution and fragmentation of both the market to include a vaccine that combines IPV vaccine with pentavalent and demand of self-financing countries.

## 3 Discussion

As an endeavour borne primarily out of the need to accelerate market development and meet public health goals, the market shaping experience for pentavalent provides valuable lessons for funders, market influencers and manufacturers on the optimal conditions, opportunities and risks of market shaping for vaccines and health products.

It took fifteen years for the market to reach relative stability, revealing the necessary long lead-times required for country adoption, vaccine development and building manufacturing capacity and to see the impact of market shaping. A more rapid evolution may have been possible with earlier implementation of active market shaping, however the timeframe is indicative of that required to drive market changes and should be planned for [43] despite the early expectations of Gavi. The assured funding of Gavi along with UNICEF and PAHO centralised procurement serve as powerful foundations and influence the design of active market shaping but are themselves insufficient to drive rapid change. While there are some unique aspects of the market for pentavalent, such as its focus in lower-income countries, the lessons and conclusions are applicable to other vaccines and global health product markets.

### 3.1 The importance of frameworks and strategies

Gavi’s evolution of supply strategies and Roadmaps provide essential frameworks to guide strategies and coordination among Gavi market shaping partners and are essential for any groups considering market shaping efforts. As demonstrated in the pentavalent case, market shaping is stronger when multiple partners contribute and coordinate actions, while seeking to prevent duplicative or counter-productive work. The evolution of the SPS, Roadmaps, PRGs and HMF to focus on the long-term and include systematic measurement of trade-offs and tensions between priorities and their consequences on market health were, and continue to be, evident needs. The HMF should be evaluated over time to confirm its benefits in communicating consistently and demonstrating areas where investments or higher prices are warranted to increase market health and those where market health is sufficient to warrant a focus on price.

Reducing information asymmetries through UNICEFs price transparency and market notes enhances partner, manufacturer and country understanding of the market. While there has been good progress in increasing mutual understanding of markets among market shaping partners, extending that understanding and a recognition of market shaper intentions to some manufacturers has been less successful. Manufacturers have cited Gavi funding as the market shaping force for pentavalent and some were less fluent in recognizing specific active market shaping intentions. This may be due to the experience of most manufacturers in the DTP market, because they did not have experience with market shaping interventions or because insufficient information was provided. Ensuring that manufacturers, who are impacted by these interventions, are adequately informed and adjusting future strategies based on feedback and results remains an area for improvement.

### 3.2 Market interventions must be adjusted to conditions

Interventions should be implemented and adjusted considering external factors and the unique needs of the market at different stages. During early market development, technical assistance for NRAs and manufacturers was essential to ensure quality and efficient regulation and stable supply at lower costs. During the next phase risk-sharing approaches and a tender that did not seek overall cost minimization were justified before demand had stabilized. These interventions, which affected multiple entities, reinforced the need for a coordinated strategy and good communication across market shaping partners. As the market matured, it was important to end risk-sharing interventions and to seek pricing at the costs of production plus a small return.

The next phase of market development, evident by 2016, deliberately shifted risk-taking to manufacturers and took steps toward reaching the stage of only active procurement. The 2017–19 UNICEF tender employed a modality that resulted in lower-than-expected pricing that is potentially lower than production costs for some manufacturers, and revealed risks related to manufacturer expectations and behaviour when experiencing a shift in responsibility for risk-taking. While manufacturers had experience with traditional UNICEF tenders, a full understanding of the intent and potential outcomes of this tender was not realized by some. The rapid evolution of the market was difficult for manufacturers to manage and a longer-term strategic, less transactional, approach to interventions is uniformly called-for from manufacturers.

### 3.3 The role of manufacturers

Manufacturers make public health impact possible and they hold responsibility for reliably producing quality, affordable, life-saving vaccines. Of the eight manufacturers with prequalified pentavalent, UNICEF is currently procuring from four, two others are available to UNICEF and two, whose products or cost structures were no longer competitive, have ceased production. After nearly a decade of market participation at higher price levels, manufacturers whose costs are no longer competitive with market pricing have exited, the gap being filled through a significant increase in capabilities from emerging manufacturers with lower cost structures. Manufacturer exits in certain market conditions are an expected part of market development and should be monitored, but not always viewed as a deterioration of market health.

Both manufacturers and market shapers of any health products hold responsibility for maintaining a partnership mentality, transparent two-way communication, and continuous learning and risk-sharing. Both parties should ensure a thorough understanding of the others point of view and each accept responsibility for their actions. Transparent information from manufacturers will help market shapers understand and work with the needs and limits of manufacturers.

### 3.4 Markets are interconnected

The actions of large funders, procurers, manufacturers and the prevailing conditions of other vaccine markets can exert significant and unpredictable external forces on a market. Prices in the Government of India tenders altered expectations for pricing and contributed to a significant decrease in pentavalent prices. While opportunities created by these forces can and should be leveraged, there is a corresponding risk that may require adjusted strategies to mitigate when seeking to maintain vaccine security and a healthy market.

Positive and negative unintended consequences of market shaping resulting from external forces and the interconnected nature of markets are likely and should be systematically measured over the long-term. The decrease of pentavalent prices for MICs could be considered a positive externality strictly from Gavi’s perspective and highlights the benefits of UNICEF’s wider remit to ensure that all MICs benefit from a competitive market. Conversely, the negative experiences of manufacturers or financial instability stemming from lower pentavalent prices should be considered within a broader context of the sustainability of vaccine markets for lower-income countries. Market shapers of any health product should have a clear strategy for managing consequences of one market’s development on others where the same manufacturers participate. Identifying and measuring unintended consequences early in market shaping implementation is ideal. In the pentavalent case, it could have been initiated earlier, however retrospective measurement remains useful to guide strategies for other vaccines.

Building the market for pentavalent was an unpredictable endeavour benefiting from activity across a range of institutions and manufacturers, multiple interventions and frequent adjustments. Lessons learned in this market should be applied to others with similar characteristics but should not be taken as absolute.

### 3.5 Case study limitations

This review is on the market for a single health product in a unique structure where the global market is dominated by three large buyers and is exclusively in low and middle-income countries. Revenue from high-income markets for pentavalent is not available to manufacturers. Results, lessons and conclusions should be taken in context.

The market has reached a state of fully satisfied demand, and thus an equilibrium in terms of supply and demand. It is, however, likely to evolve to incorporate a combination vaccine including IPV. This may essentially reset the market parameters equivalent to the development of the pentavalent market from its beginning, requiring ongoing strategy adjustments.

While the practice of market shaping remains in an early stage, those engaged in this field for global public health products should establish systems for monitoring long-term intended and unintended consequences of market shaping and should continue to publish methods, results and lessons.

## 4 Conclusions

**Fig. 8:**
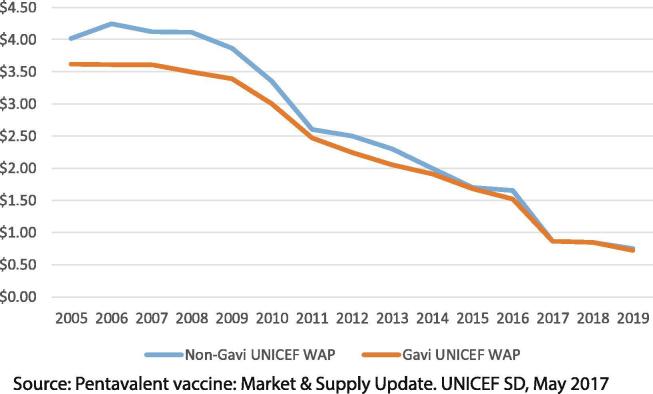
Weighted Average Price (WAP) through UNICEF for Gavi-supported and other countries, 2005–19.

Outcomes in the pentavalent market provide strong evidence of the benefits of market shaping including an ability to vaccinate nearly 80 M infants in lower-income countries per year against five diseases at a vaccine cost of ∼US$2.50 per child. Supply is sourced from a diversified base of manufacturers with relatively low technical risk and vaccines satisfy customer needs. Donors and countries save >US$500 M annually when procuring pentavalent when compared with 2010 prices (Fig. 6, Fig. 8). Broader success of Gavi’s market shaping strategy is supported by results showing that in 2017, eight vaccine markets had supply sufficient to meet demand, against the goal of 11 vaccine markets with sufficient supply by 2020 [44].

Market shaping partners acknowledge that benefits are balanced by potential risks [3], [5] resulting from interventions in a highly consolidated market. The unintended consequences of market shaping include altering manufacturer investments in new products or ceasing production of existing products and the potential for negative pricing consequences for other vaccines or countries [4], [5]. While the pentavalent results are beneficial for countries and donors, prices of ∼US$2.50 per course (∼US$0.85 per dose) raise concerns among all stakeholders about the financial sustainability of pentavalent manufacturing and supply, potentially constraining investment in next generation vaccines. The market requires time to stabilize and ongoing careful management, specifically with respect to vaccine security, which should remain at the heart of market health considerations.

As a major funder of vaccine procurement, Gavi’s Board should maintain market shaping as a strategic focus of Gavi. It should evolve the strategy with the needs of countries and manufacturers, and provide enough resources for its planning and implementation, and recognize that it will take significant time to see benefits. The Gavi Board should recognize the balance required between the tensions inherent in market shaping and should support choices that create “healthy markets” and keep vaccine security central, even when not the lowest-cost option.

Other funders and groups that engage in health product market shaping should do so based on an inclusive, high-level endorsed strategy and a framework for clearly describing the desired outcomes (i.e., “healthy market”). They should engage manufacturers as partners, acknowledge the benefits, limitations and risks of market shaping, establish a mechanism for measuring unintended consequences and publish best practices. Case studies like this one, focused on markets with other characteristics such as widespread global use of the same health product or markets for regional diseases, would be welcome additions.

Health product manufacturers should benefit from market advances and contribute to market shaping. They should be informed and responsible partners and participants, understanding the intent of market shapers and limits of the market shapers scope of control. They should use published information, alongside their own, to understand customer preferences and the competitive landscape. While market shapers seek to reduce information asymmetries and sometimes to reduce risk for manufacturers, ultimately manufacturers must independently assume responsibility for their actions and assume risks within each of their capabilities.

A goal of market shaping is to help lower-income countries achieve and sustain public health and development gains through increased access to affordably priced, suitable health products. To leverage these conditions, self-financing countries should strategically manage health product procurement, including an analysis of the potential benefits of using a pooled procurement/group purchasing mechanism. Countries should also utilize market intelligence to inform procurement and engage as stakeholders through providing information (e.g. through the WHO’s Market Information for Access to Vaccines database for vaccines and/or peer networks such as the Vaccine Procurement Practitioners Network) that can reduce information asymmetries.

The actors, structures and methods employed in market shaping will need to adapt to the changing global health landscape and innovate new ways of working, while building on evidence of successful strategies. A future marketplace with less centralised procurement will require new strategies. Market shapers must also accept that consequences of their actions are not limited to the intended vaccine market and will likely have effects on others where the same manufacturers participate.

Even the pentavalent story is far from complete. The market is entering a transition with the risks and opportunities of splitting or being replaced with an IPV-containing combination vaccine. The challenge will be to maintain vaccine security and market health – including stable supply and sustainable prices of pentavalent -- while encouraging innovation and development of an affordable combination with IPV vaccine. The roles and need for action on the part of the manufacturers and market shapers are not over; it is time to continue adapting strategies to meet new market conditions, using collaborative and transparent methods and ensure that future generations will benefit from resulting investment and market dynamics.

## Author contributions

MM, GG, and RI participated in the conception and design of the case study; MM, EB, GG, AJ, PK, YM, AN and RI participated in drafting or revising the article for intellectual content and all authors approved the final version to be submitted.

## Declaration of Competing Interest

The authors declare that they have no known competing financial interests or personal relationships that could have appeared to influence the work reported in this paper.
